# Thinking “ethical” when designing an international, cross‐disciplinary biomedical research consortium

**DOI:** 10.15252/embj.2020105725

**Published:** 2020-09-07

**Authors:** Maria‐Elena Torres‐Padilla, Annelien L Bredenoord, Karin R Jongsma, Astrid Lunkes, Luca Marelli, Ines Pinheiro, Giuseppe Testa

**Affiliations:** ^1^ Institute of Epigenetics and Stem Cells (IES) Helmholtz Zentrum München München Germany; ^2^ Faculty of Biology Ludwig‐Maximilians Universität München Germany; ^3^ Department of Medical Humanities Julius Center University Medical Center Utrecht Utrecht The Netherlands; ^4^ Institute of Functional Epigenetics Helmholtz Zentrüm München München Germany; ^5^ Life Sciences & Society Lab Centre for Sociological Research KU Leuven Leuven Belgium; ^6^ Department of Experimental Oncology IEO, European Institute of Oncology IRCCS Milan Italy; ^7^ Department of Medical Biotechnologies and Translational Medicine University of Milan Milan Italy; ^8^ Nuclear Dynamics Unit Institut Curie PSL Research University Paris France; ^9^ Department of Oncology and Hemato‐oncology Università degli Studi di Milano Milan Italy; ^10^ Human Technopole Milan Italy

**Keywords:** Molecular Biology of Disease, S&S: Ethics

## Abstract

This commentary outlines challenges with identifying and implementing ethical, legal and societal considerations when initiating large‐scale scientific programs and suggests best practices to ensure responsible research.

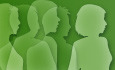

Understanding and measuring the impact of decisions in research, or the implementation of a research project and new technologies is not straightforward. While research communities have become increasingly aware of the ethical, social and legal implications of the research they conduct, it is more challenging to anticipate such consequences arising from complex, highly interdisciplinary research projects involving multiple sides and technological components. Thus, there is a strong need, especially given the rapid rate of technological advancement, to anticipate, prepare and implement measures that enable responsible research. This challenge is particularly important when designing research programmes involving large‐scale consortia as the European LifeTime initiative spanning multiple countries and institutions.

This article will comment on two major aspects. Firstly, we will discuss topics that have emerged as ethical implications of LifeTime. These implications do not cover all the breadth of the research covered by LifeTime, but are particularly relevant to our current society and include (i) application of artificial intelligence to health care, (ii) patient consent and (iii) data protection, particularly in the context of disease diagnosis. The second aspect proposes strategies that will be applicable to any consortium of a similar kind, with concrete mechanisms for engaging with ethics. These include (i) the realization of an initial interdisciplinary symposium, (ii) a yearly roadmap for follow‐up meetings to evaluate progress and (iii) a strong focus on public engagement, for example through arts. A key overarching recommendation is that engagement of biomedical research with ethics is not a “one off” step, but a continuous process involving interdisciplinary individuals throughout the consortium, for example under an Ethics Parallel Research mechanism.

Science and technology contribute to societal goals in many regards, from understanding basic biology to enhancing scientific training and advancing medicine. The potential for preventing and mitigating illness is increasingly believed to lie at the cellular level. Therefore, the LifeTime Initiative (https://lifetime-fetflagship.eu/) was launched in 2018 with the primary aim of studying early mechanisms of cell alteration during disease progression to enable early diagnosis and disease interception. LifeTime aims to do this by integrating single‐cell multiomics, patient‐derived organoids and machine learning to transform the precision of health care at a sustainable, patient‐relevant scale (Rajewsky *et al*, 2020). These three axes of technological innovation constitute a major drive for current research, and we anticipate that the results generated have the potential for tremendous breakthroughs to improve quality of life throughout the disease course.

With the LifeTime initiative involving researchers from over 20 countries in Europe, one of our main tasks is to help identify the main risk areas for implementing ethically responsible, socially robust, and legally compliant research, and anticipate solutions and preventive measures. To enable the implementation of such large‐scale projects, ethical, legal and societal issues have to be identified, evaluated and considered from the initial stages of the project, so that they shape the final experimental and clinical design. Here, we describe our experience in setting up an Ethics & Society pipeline in a large biomedical research consortium.

## Ethics engagement: Ethics Parallel Research

Ethics engagement can take different formats, from *ad hoc* consultation with ethicists to ethics teams working alongside or embedded within research teams (Sugarman & Bredenoord, 2020; Even Chorev & Testa, 2020). The responsible development of the research and the converging technologies developed in LifeTime should utilize a prospective ethics mechanism, that is “ethics from within” (Kudina & Verbeek, 2018), rather than a retrospective, and thus, reactive approach. For this, a comprehensive strategy to expand the scope of bioethics from clinical actions and practices to the anticipation and embedding of normative questions within the design of a biomedical project is required. One approach that has proven suitable for the proactive, constructive study of novel biomedical technologies is Ethics Parallel Research (Sugarman & Bredenoord, 2020; Jongsma & Bredenoord, personal communication). Here, ethicists identify and evaluate the ethical challenges associated with a novel biomedical technology in parallel or even proactively as the field progresses—rather than once the technology is fully developed. The advantage of this ethics parallel method is that it promotes ethical reflection and guideline making from the beginning. This allows the “co‐production” of technological and normative innovations with the relevant stakeholders (Jasanoff, 2004). Indeed, if ethicists, (bio)medical scientists, lawyers, patients and other relevant stakeholders are involved in a consortium from the beginning, normative visions and experimental design can be confronted and integrated in ways that reflect, from their conception, the two defining aspects of human ingenuity: changing the world and making sense of it.

## LifeTime lessons: technology and society

LifeTime aims to understand the onset and progression of complex diseases and their response to therapy at single‐cell resolution. During the preparatory phase of LifeTime, we became increasingly aware that conducting research to fulfil such objectives would prompt ethical and societal implications. To identify such possible implications, we organized the “LifeTime Ethics Workshop” at the Helmholtz Centre Munich in July 2019, with a broad panel of specialists acknowledged below. Specifically, through this discussion we aimed to shed light on whether and how to implement structures and set up specific committees in order to identify ethical challenges and achieve best practices.

During this workshop, we concluded that some of these questions were as follows. What are the potential risks and benefits of introducing artificial intelligence into the clinics? How can scientists and clinicians be encouraged to take into account the ethics of artificial intelligence and organoids? How do we ensure patient data sharing while securing patient privacy? How can researchers and institutions engage the public more effectively and promote trust in science and governments in a two‐way dialogue?

This commentary does not intend to provide a solution to all of the questions raised. Instead, it is meant to reach out to the community to promote an attitude of proactive reflection by everyone involved, and to suggest how such large‐scale initiatives could adopt an ethics mechanism. In addition, we discuss below a few specific points that can be taken into practice when designing a new research consortium of this calibre.

## Main ethical and societal considerations

The advancements achieved with innovative technologies in prevention and diagnosis impact the way our society perceives and understands health, illness and therapeutics. For example, the availability of a new treatment poses the question to the society of who should have access to it and to the affected people of whether they want to receive it. It also raises questions of health and illness, because the notion of normality or health shifts over time and is likely to continue shifting with the advances in interception medicine that LifeTime will enable. Research findings will also likely raise questions of equity and non‐discrimination, for how do we prevent stereotyping based on an early diagnosis, which will potentially limit the choices and participation in society of those individuals who are affected, far before potential onset of disease? If early detection and/or continuous monitoring become the norm, how do we ensure that patients who do not wish to know if they will develop a certain disease will have the same rights to medical care upon disease onset or worsening? Personalized medicine also raises questions of responsibility, accountability and affordability.

## Some strategies to set in place

### A need for continued interdisciplinary dialogue

A fruitful way to find adequate means to address these questions is to set in place mechanisms that enable an interdisciplinary, continued dialogue with ethicists, lawyers, philosophers, economists and other social scientists, as well as patients’ organizations from a very early stage of the project, and importantly throughout its implementation. Concretely, we suggest an initial symposium to identify such aspects and related risks and implications, and establish priorities and Task Force groups to address them. This initial meeting will also enable the identification of areas in which the consortium can provide unique contributions to solve particular issues, which will only be possible through the concerted effort of multinational participation. Such meetings also provide valuable opportunities to improve mutual understanding between the many different disciplines involved. Further scholarly research in the humanities (ethics) and social sciences is needed, sometimes combined with empirical research, to reach ethically and socially sound conditions and recommendations.

Continuous monitoring, in the form of yearly consortium meetings, should be tasked with the evaluation of emerging ethical and societal aspects from new technology or its application. The annual consortium meetings should also seek to identify current weaknesses or areas where progress was inadequate and take actions to pinpoint areas of expertise, which are missing, and call *ad hoc* experts accordingly. The Task Forces formed in the initial meeting should meet regularly so that concrete approaches can be formulated for patient groups, researchers or data collectors, as appropriate.

It seems essential to maintain an interdisciplinary group of people and bring these specialists to the same table. The goal is to promote a continuous and open dialogue that evaluates possible scenarios, and provides concrete applicable advice to policymakers and governmental bodies, hand in hand with the development and execution of the research plan.

### Seek continued communication with the public: thinking and communicating through Art

In addition to a workshop and regular meetings within the consortium, another key aspect is to seek a thorough and constant communication with the public through public meetings, citizen panels and Civil Society Organisations. In fact, technologies and scientific research will impact and affect social reality, and therefore, the public has stakes in the involvement and co‐production throughout the research project and should not be surprised with a technological innovation *ex post facto*. We should always bear in mind that knowledge and technologies impact multiple scales, from the individual to the societal level, with the potential to narrow or widen social inequalities or enact discriminations of different sorts. We must therefore anticipate potential harmful avenues in the use of new technologies, to pre‐emptively envisage and prevent the potential negative individual and societal implications of research.

We must also make sure that researchers create an open and inclusive environment for societal dialogue. Clear strategies need to be sought and implemented in order to establish a transparent dialogue with the public about technologies and research. Information related to the technology, its use and objectives therein must be accessible and understandable. Ideally, society at large should be involved in the development of technologies in order that they have ethical and social legitimacy. The “Communication” and the “Training and Education” programmes of LifeTime in this case should implement the development of communication strategies (Rajewsky *et al*, 2020). These include website and newsletters, participation in podcasts and interviews in various media outlets, and the organization of outreach events to enable such dialogue with the public.

Fruitful and concrete examples of more creative strategies are illustrated by the collaboration between research institutions or universities and artists (see, for example, Science Gallery International). Art can provide a powerful and unique way to engage with and communicate with the public. The goal of communicating through artistic endeavour in this context is to inspire people through their lived experience to think about possible scenarios and to promote individual critical thinking. Keeping the balance between promise‐making towards lay public and being realistic with what research can deliver should also be an integral part of the research endeavour for which arts and dedicated communication tools can be helpful. Going beyond classical communication with lay public, and integrating critical thinking of public in our approach, will help to transform and provide additional input for conducting responsible research and build trust. A concrete recommendation here is implementation of Artists in Residency programmes within the consortium. Biomedical research consortia should dedicate part of the budget for these kinds of activities.

### Training: creating awareness in the next generation

Another key recommendation from our workshop is the establishment of ethics modules for training the younger generation as well as acting clinicians and researchers to make them aware of the societal implications of research and the use of technology. This can be organized through a training programme, which should contain the priorities established in the initial workshop mentioned above, through dedicated modules. In the case of LifeTime, these would include an Education Module on “Ethics of applied Artificial Intelligence” and on “Data Protection”, for example. The design of the training modules should be done jointly with the working taskforces of the LifeTime “Training and Education” programme. The objective would be to make a working group, which should have a presence in individual countries of the consortium, and ensure that these training activities are funded and take place accordingly.

## Some points to consider: potential ethical and legal implications in LifeTime

### Data governance

The enactment of the EU General Data Protection Regulation (GDPR) in 2018 served as a cornerstone for the new data governance framework of the EU, with the goal of harmonizing data processing operations across the continent (Marelli & Testa, 2018). However, Member States have still retained prerogatives to legislate over a number of key aspects—such as the processing of health‐related data—leading to the progressive implementation of different national data protection regimes in the health sector. Likewise, data governance and ethics oversight, carried out by national data governance bodies and local ethics and data access committees, are still prevalently anchored at the institutional level and are subject to national (and sometimes even regional) regulations.

This creates a notable challenge for large‐scale consortia of LifeTime's ambition and reach. A key, paradigmatic challenge revolves around consent. Consent procedures still eschew harmonization across Europe and are not always agile enough. In addition, ethics and data protection requirements may diverge. Namely, consent may represent a necessary prerequisite for enrolling participants in research but not a required legal basis for the processing of personal data.

Vis‐à‐vis such complexity, international consortia should be instrumental in implementing harmonized procedures underpinning ethically sound data sharing, while also channelling insights and recommendations coming from a varied set of stakeholders through to policymakers.

### The consent: the patient, the clinician and the researcher

Discussions over consent attracted a lot of attention during our workshop. Research ethics committees are in place in most European (academic) hospitals and act in accordance with local, national and international regulations. Often though, the guidelines or timelines for each committee are not harmonized with those of other institutions, notably across different countries. Therefore, a recommendation that emerged was the creation of Task Force group specifically focusing on establishing procedures to harmonize fragmented procedures, by developing (amongst others) a consent form, with a patient‐centred view in straight dialogue with the treating clinicians, research ethics committees and the researchers involved in the consortium. Ideally, this should be done at a multinational level and, most importantly, should facilitate the involvement of patients and patient advocates in the development of consent and governance procedures. An ongoing conversation with the patient and the physicians involved should be maintained, while balancing the potential burden of the patient's involvement. Globally, human subject research, such as that conduced in LifeTime, should provide a centre stage to patients and include their interests at the heart of the research programme. Notably, researchers must remember that within research projects that entail the collection of human data, data subjects always retain ownership of their data as well as important rights and prerogatives of control over the data. The consent is therefore crucial to enable a transparent, fair and legal use of the data.

### Artificial Intelligence and health care

Artificial intelligence (AI) has great potential for improving healthcare decision‐making. Besides potential tensions with data protection principles and mechanisms deriving from the implementation of AI technologies (Marelli *et al*, 2020), important points of consideration here are the actual input data used to train AI applications and the labelling and the algorithms used to analyse the data. One of the major potential risks with applying AI in a medical context concerns potential bias and discrimination. For example, if certain groups have been excluded from data collection, e.g. if only data of Caucasian individuals are used to train AI applications, the model cannot be generalized to other groups. This may not only result in wrongful application of such models for individuals they are ill‐suited for, but may also discriminate against the excluded groups in terms of appropriate and equal access to care. It is therefore imperative to ensure that representative and inclusive datasets derived from high‐quality research are used for developing AI‐based solutions that consequently are widely applicable. Therefore, funds for research must be carefully planned for inclusive implementation of AI, including the development of AI methods, applications and algorithms that are not biased. It is also essential to keep in mind that the final decision‐making, even if it is aided by an AI approach, must pertain to the subject (e.g. the treating physician in this case).

### Equity and access

Access, equity and diversity also emerged as important points during the LifeTime Ethics Workshop. Large consortia should also work towards equity of access to health care. The free flow of data between European countries should help to promote the right of access to technologies of diagnosis independent of the country of residence. However, additional complications stem from the differences in health care and reimbursement systems. Ideally, LifeTime, as a model for multinational consortia, should design and apply a roadmap for the inclusive implementation of regulated data access within Europe. This could be done by the participation of LifeTime protagonists in decision‐making bodies and through the sharing of a “pilot” governance model structure and data access committees.

## Conclusions

Overall, working towards societally impactful research requires addressing ethical, societal and often also legal issues throughout the research process. Innovation should be socially embedded and responsive towards needs and input from society. Researchers need to reflect on the social impact of the envisioned novel technologies. To achieve all these, it is essential to create a forum to empower a continuous examination and co‐production of the research objectives by ethics, law and communication experts. We suggest the early establishment of a symposium with emerging working groups to identify ethical implications and potential risks, solutions and implement a continued evaluation throughout the duration of the project.

The combination of arts, science and humanities can help to understand the complexity of large consortia involving different cultures, expectations and infrastructures beyond political borders. They should help us to transcend the objectives of a consortium like LifeTime by developing awareness of researchers and the public in general. Engagement with ethics, as well as values such as equity, diversity and the integration of these into the scientific process and project design should be a priority. We currently hold an outstanding innovation opportunity, whereby consortia such as LifeTime could be European role models as pioneers in collaborating across scientific boundaries, and use ethics as driver.

## Conflict of interest

The authors declare that they have no conflict of interest.
